# Metabolic modelling in a dynamic evolutionary framework predicts adaptive diversification of bacteria in a long-term evolution experiment

**DOI:** 10.1186/s12862-016-0733-x

**Published:** 2016-08-20

**Authors:** Tobias Großkopf, Jessika Consuegra, Joël Gaffé, John C. Willison, Richard E. Lenski, Orkun S. Soyer, Dominique Schneider

**Affiliations:** 1 School of Life Sciences, University of Warwick, Coventry, UK; 2 University of Grenoble Alpes, Laboratoire Techniques de l’Ingénierie Médicale et de la Complexité - Informatique, Mathématiques et Applications, Grenoble (TIMC-IMAG), F-38000 Grenoble, France; 3 Centre National de la Recherche Scientifique (CNRS), TIMC-IMAG, F-38000 Grenoble, France; 4 University of Grenoble Alpes, Institut de recherches en technologies et sciences pour le vivant – Laboratoire de chimie et biologie des métaux (iRTSV–LCBM), Grenoble, F-38000 France; 5 CNRS, iRTSV–LCBM, F-38000 Grenoble, France; 6 Commissariat à l’énergie atomique (CEA), iRTSV–LCBM, F-38000 Grenoble, France; 7 Department of Microbiology and Molecular Genetics, Michigan State University, East Lansing, MI 48824 USA; 8 BEACON Center for the Study of Evolution in Action, Michigan State University, East Lansing, MI 48824 USA

**Keywords:** Adaptive diversification, Experimental evolution, FBA, *In silico* evolution, Tradeoffs

## Abstract

**Background:**

Predicting adaptive trajectories is a major goal of evolutionary biology and useful for practical applications. Systems biology has enabled the development of genome-scale metabolic models. However, analysing these models via flux balance analysis (FBA) cannot predict many evolutionary outcomes including adaptive diversification, whereby an ancestral lineage diverges to fill multiple niches. Here we combine *in silico* evolution with FBA and apply this modelling framework, evoFBA, to a long-term evolution experiment with *Escherichia coli*.

**Results:**

Simulations predicted the adaptive diversification that occurred in one experimental population and generated hypotheses about the mechanisms that promoted coexistence of the diverged lineages. We experimentally tested and, on balance, verified these mechanisms, showing that diversification involved niche construction and character displacement through differential nutrient uptake and altered metabolic regulation.

**Conclusion:**

The evoFBA framework represents a promising new way to model biochemical evolution, one that can generate testable predictions about evolutionary and ecosystem-level outcomes.

**Electronic supplementary material:**

The online version of this article (doi:10.1186/s12862-016-0733-x) contains supplementary material, which is available to authorized users.

## Background

The ability to predict evolution would be valuable not only for understanding such processes as adaptation and speciation [1–3], but also for engineering robust industrial strains, anticipating ecosystem responses to climate change, and combatting antibiotic resistance [4–7]. Models that capture the relationship between genotypes and environments, the structure and state of regulatory and metabolic networks, and the resulting phenotypes are likely to be important for developing these predictive abilities [1, 3, 8]. Ultimately, models of the relationship between genotype and phenotype will need to be combined with models of evolutionary and ecological dynamics in integrated frameworks that can predict the trajectory of evolution [5, 9].

The dynamics of evolutionary change reflect multiple processes and varying selective pressures that are influenced by many ecological, physical, and cellular constraints that may conflict with one another. Understanding whether and how these dynamics lead to the splitting and divergence of lineages is of central interest, as these processes represent the initial steps towards speciation. To this end, several theoretical studies have shown that cellular tradeoffs can promote lineage divergence [10–16]. The importance of such tradeoffs can be readily understood in the context of metabolism and growth. For example, if there were no tradeoffs, then one would predict that cells should maximize their expression of transporters and their surface area to achieve the highest possible rate of substrate uptake [17]. However, such cellular investments would impinge on other cellular processes owing to competing requirements for membrane and cytosol space [18, 19], ribosomes [15, 16], and redox carriers [20, 21]. Thus, cells may appear suboptimal for individual physiological parameters, but this might be merely a consequence of being optimal for the combined set of parameters and associated cellular tradeoffs.

Historically, the interplay between cellular tradeoffs and evolutionary and ecological dynamics has been analyzed using game theory and differential equation-based models that consider small or idealized metabolic systems [10, 11, 14, 22]. These studies have highlighted that tradeoffs in cellular metabolism can lead to incomplete degradation of a resource, resulting in the evolution of cross-feeding interactions [10, 11]. This phenomenon has been seen in several evolution experiments under both batch and chemostat conditions [23–27]. To increase predictive power in microbial ecology and evolution, it is now desirable to develop models that can take into account cellular metabolism at a larger scale and across different organisms. Stoichiometric models offer a promising approach because, in principle, they can capture all enzyme-mediated metabolic reactions of an organism in an unbiased and non-supervised way using genomic information [8].

Flux Balance Analysis (FBA) has been developed to determine the optimal metabolic state of an organism, given knowledge of its biochemical network, biomass composition, and uptake flux rates [28]. This approach is based on the assumptions that evolution has optimized metabolism and that metabolic fluxes can be predicted by setting the growth rate for a given rate of substrate uptake (such that the ratio of the two rates represents a yield) as an optimization criterion that can be solved by linear programming [28–30]. Early applications of FBA ignored the essential role of tradeoffs in the computation of metabolic fluxes [28, 31, 32], but more recent applications have incorporated tradeoffs as constraints on total fluxes [18, 19, 33, 34] and thereby achieved better prediction of experimentally observed metabolic states, such as preferential substrate utilization [19] and acetate overflow [18]. Experimentally measured reaction thermodynamics and gene expression levels have also been used to constrain optimal metabolic states that reflect tradeoffs [35–37], and there have been efforts to combine FBA with ecological interactions between multiple species in microbial communities [38–45]. These approaches use species-specific models in a shared environment to maximize a predefined, community-level objective [39, 41, 43, 44] or apply FBA within a dynamic framework [46]. The latter approach enables prediction of ecological interactions such as competition and cross-feeding between different species making up the model community, given defined substrate uptake constraints for each model species [40, 42, 45]. However, none of these approaches can currently be used to predict the interplay between ecological and evolutionary dynamics.

Here, we begin to overcome these limitations by integrating a FBA model of multi-phenotype systems with both cellular constraints and evolutionary dynamics. We define an overall constraint on uptake rates to enforce tradeoffs while simulating multiple model organisms living in the same environment without the need to specify each organism’s uptake preferences a priori (for details on how evolution and mutations are simulated see Methods section). By limiting total uptake in the model, and including O_2_ “uptake” in that total, we seek to represent cellular limitations that can arise from many diverse processes, including redox cycling [20, 47], respiratory chain [18], enzyme expression [16, 48], and substrate uptake [17]. Although O_2_ uptake *per se* might not be limiting, limitations in the electron transport chain can effectively limit O_2_ respiration. Accounting for all the different possible limitations arising from cellular processes in a mechanistic manner is beyond the scope of stoichiometric models; however, limiting total uptake provides a general constraint that allows us to implement the tradeoffs observed in different studies in a simple, consistent, albeit approximate manner [16–18, 20, 47]*.*

This approach allows integration of evolutionary dynamics by mutations that change substrate uptake rates along with the optimization of each model organism in the context of other model organisms that are present and coevolving in the same environment. The combined framework, which we call evoFBA, thus aims to provide a more realistic way to model the interplay between ecological and evolutionary dynamics with global constraints arising from cellular tradeoffs. To the best of our knowledge, this is the first FBA modeling approach that captures the continuous adaptation of organisms to the interplay between ecological and evolutionary dynamics in systems with multiple strains or species.

To examine the ability of evoFBA to capture ecological and evolutionary dynamics, we used it to simulate the evolution of *Escherichia coli* populations in a defined glucose-limited environment with daily transfers. We then experimentally analyzed the predictions of evoFBA in the context of the long-term evolution experiment (LTEE) with *E. coli*, in which 12 populations started from a common ancestor have been propagated in a glucose-limited medium for more than 60,000 generations [2, 49]. We found that the evoFBA simulations predicted the emergence of cross-feeding model organisms as a stable end-point, which in fact has occurred in at least one of the LTEE populations [26, 50]. Moreover, we saw that key metabolic features of the model organisms were in qualitative agreement with the physiological properties we measured for the two biological lineages that emerged and subsequently coexisted for more than 50,000 generations.

## Results

Microbial communities and their underlying metabolic interactions reflect the ecological and evolutionary histories of the component species [51]. To capture these interactions, we combine stoichiometric metabolic models with ecological and evolutionary dynamics in the multi-layered evoFBA framework (see Methods). To test the utility of this framework, we apply it to the LTEE in which *E. coli* populations evolve in a defined glucose-limited environment [2, 52].

To model the LTEE, we ran evoFBA simulations starting with a metabolic model of *E. coli* that accounts for 14 carbon sources including glucose and byproducts that can be scavenged from the environment to produce biomass and fuel associated core metabolic reactions. In each evoFBA simulation, we allowed the metabolic model to change by random mutations under global constraints that must be obeyed. Thus, each simulation produced mutant model organisms exhibiting different uptake rates, metabolic flux patterns, and resulting growth rates.

### evoFBA predicts evolution of cross-feeding between lineages with different metabolic flux distributions

**Fig. 1:**
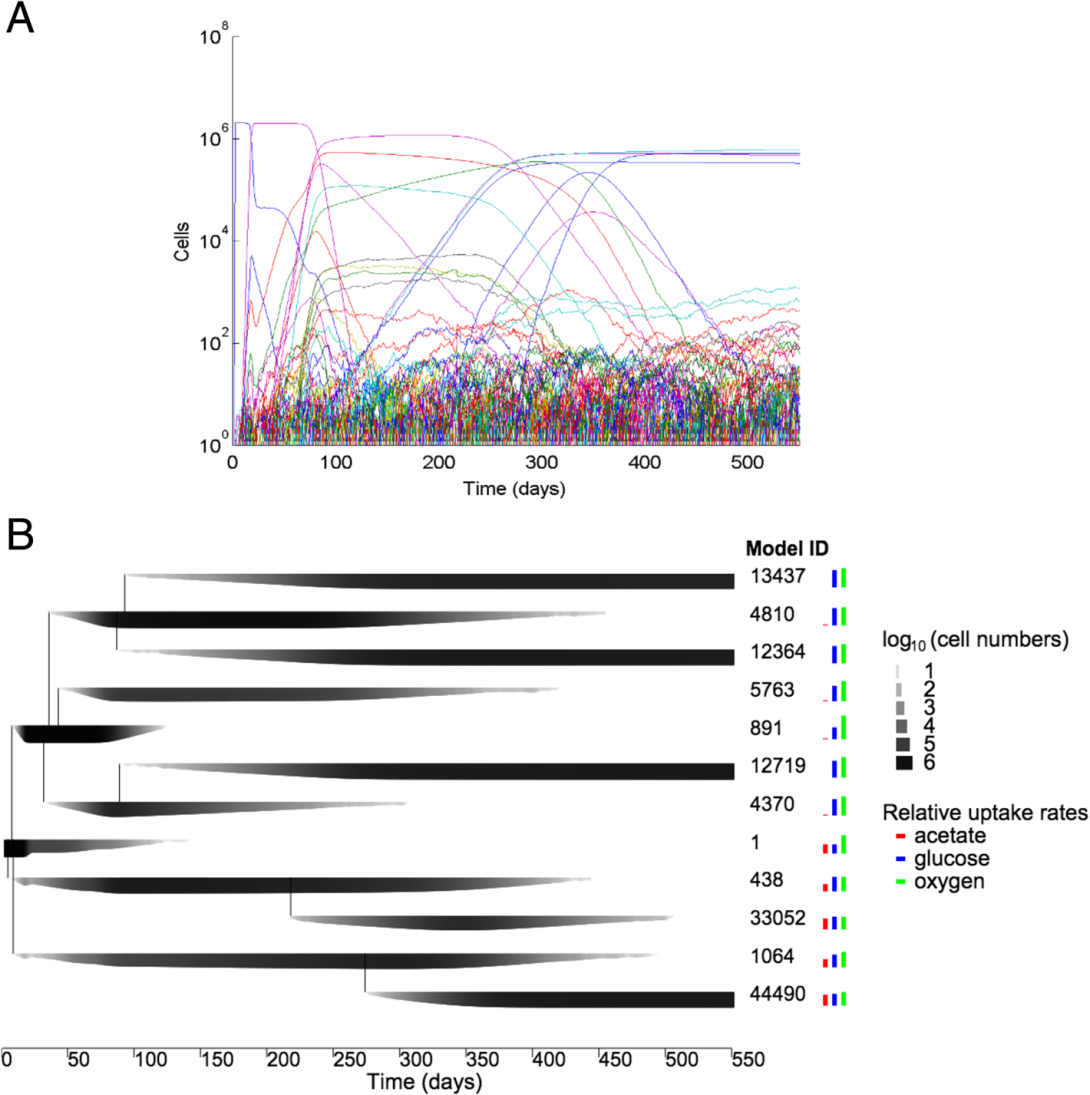
Evolutionary dynamics *in silico*. **a** Numbers of surviving cells (i.e., post dilution) after each simulated cycle on a logarithmic scale. Each curve shows one of the 3943 model organism genotypes that survived at least one cycle (see text). **b** Relationships among ancestral and mutant model genotypes for those that reached a population of at least 10^5^ cells at any point during the simulation (see Methods). Model ID indicates the identifier assigned to each model genotype, with 1 being the ancestor. Line thickness is proportional to the log_10_-transformed number per 10-ml volume at the start of each cycle. Coloured bars show relative uptake rates for glucose (*blue*), acetate (*red*), and oxygen (*green*)

**Fig. 2:**
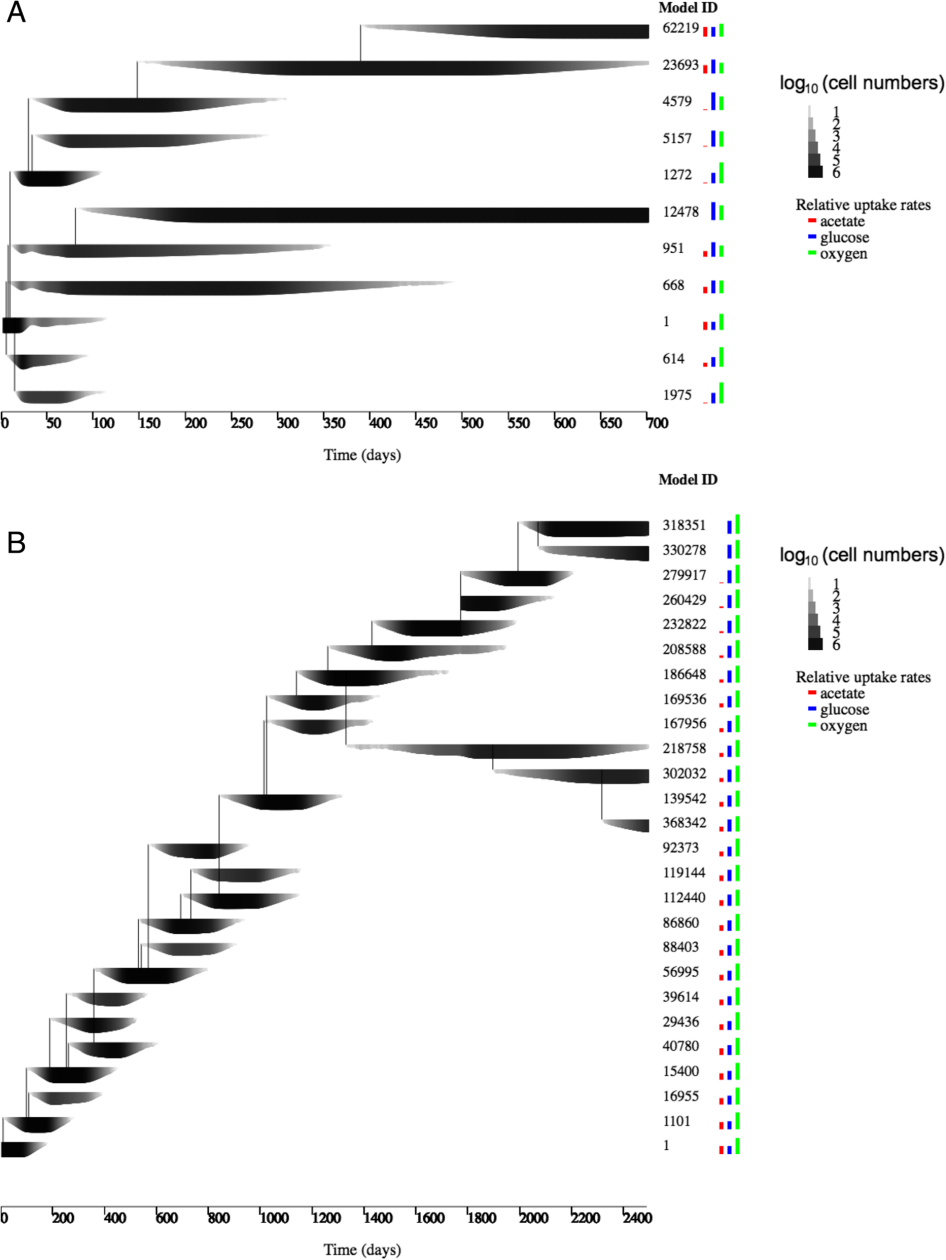
Replicate runs of evoFBA. **a** One of five replicate simulations using the same parameter set as described in the main text and shown in Fig. 1. All simulations led to qualitatively similar outcomes. **b** Running evoFBA simulations with a smaller maximum mutation step size (+/ −1 mmol/gDW/h), see Methods eq. 5), led to the same diversification into glucose specialist and glucose-acetate co-utilizing model organisms, although the time required to achieve the diversification was substantially longer. Model ID, line thickness and coloured bars are the same as in Fig. 1

**Fig. 3:**
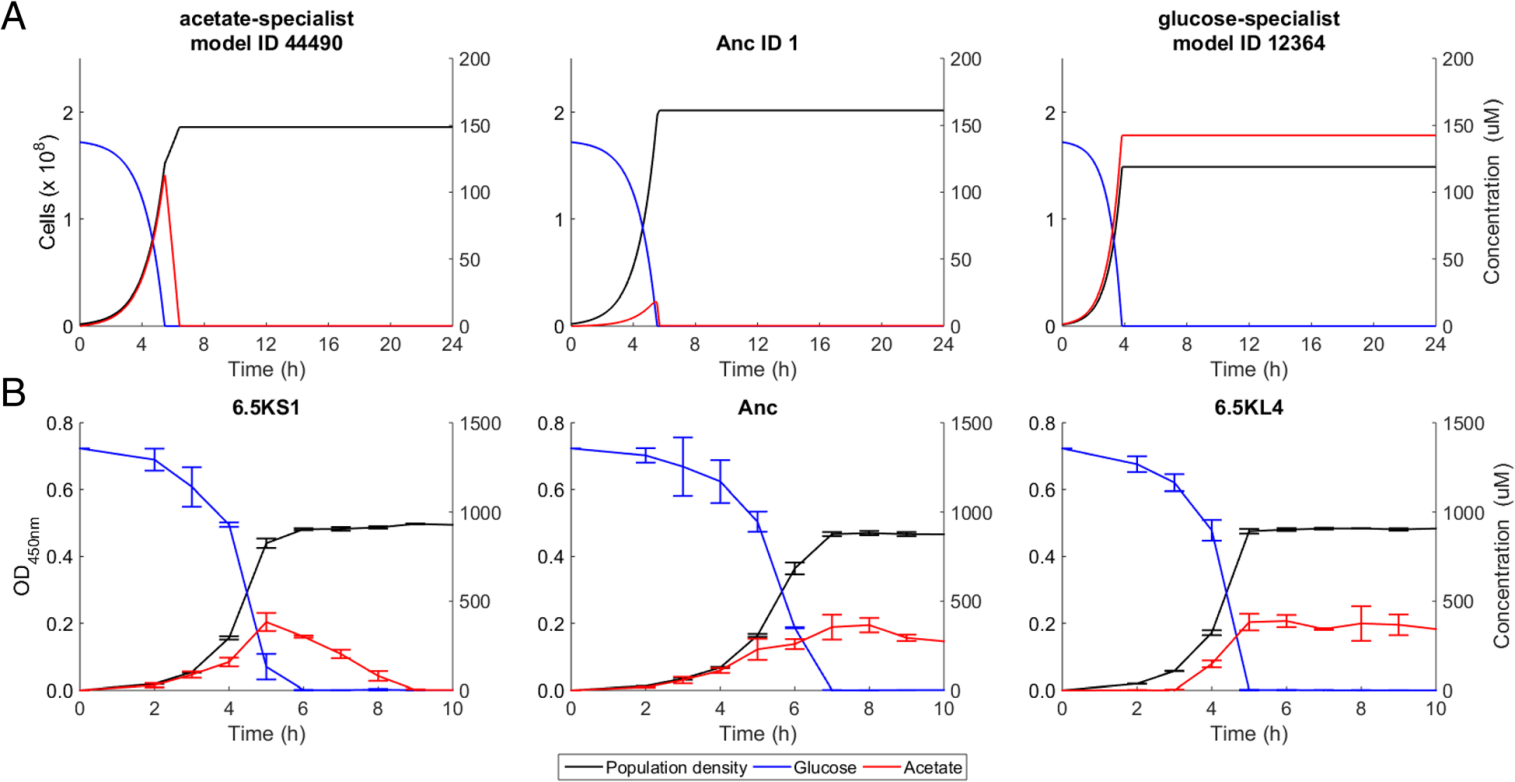
Simulated and experimental dynamics of population density and substrate concentrations. **a** Simulated dynamics over a 24-h transfer cycle for the evolved acetate specialist (*left*, ID: 44490), ancestral (*middle*, ID: 1), and evolved glucose specialist (*right*, ID: 12364) model organisms. Model IDs are the same as in Fig. 1b. **b** Experimental data for the 6.5KS1 (*left*), ancestral (*middle*), and 6.5KL4 (*right*) clones from the LTEE. Biological experiments were performed at a 10-fold higher concentration of glucose than the simulations to increase cell density and thereby improve the accuracy of the measurements of cell growth and concentrations of residual glucose and secreted acetate. Biological data are means of three replicate cultures and error bars show standard deviations

Starting from a population of identical model organisms under conditions similar to the LTEE, a typical evoFBA simulation produced through random mutations more than 90,000 genetically distinct model organisms over 550 simulated daily transfer cycles (Fig. 1). The evolutionary dynamics across replicate simulations were highly reproducible in their key features, in particular the diversification of the population into two coexisting lineages (Fig. 2). Thus, throughout the paper, we will focus on results from a typical representative simulation that resulted in 97,912 different model genotypes, of which 3943 survived at least one transfer event (Fig. 1a) and 12 reached a population size of at least 10^5^ cells at some point (Fig. 1b). These simulations revealed specific changes in oxygen, glucose, and acetate uptake by the model organisms (Fig. 1b). Glucose uptake and incomplete oxidation resulted in acetate secretion by the ancestral model organism, which would then switch to acetate uptake and oxidation after the glucose was exhausted. Thus, the ancestral model displayed a diauxic shift (Fig. 3a), as observed in *E. coli* [21]. As the *in silico* evolution proceeded, new model organisms arose that had increased glucose uptake and acetate production. The resulting increase in acetate concentration generated an ecological niche that was colonized by other model organisms with increased acetate uptake but reduced glucose uptake. After ~300 simulated daily transfer cycles (~2000 generations), the simulated evolution came to a halt, with no mutant model organisms able to replace the dominant ones. Thus, the *in silico* dynamics produced two distinct lineages that specialized on glucose and acetate, respectively. The glucose-specialist model organisms lost the ability to consume acetate, whereas the acetate-specialist model organisms retained the ability to consume glucose but at a lower rate, and the timing of their diauxic shift was changed (Fig. 3a). As a consequence, the simulation led to a stable cross-feeding relationship between two lineages of model organisms.

**Fig. 4:**
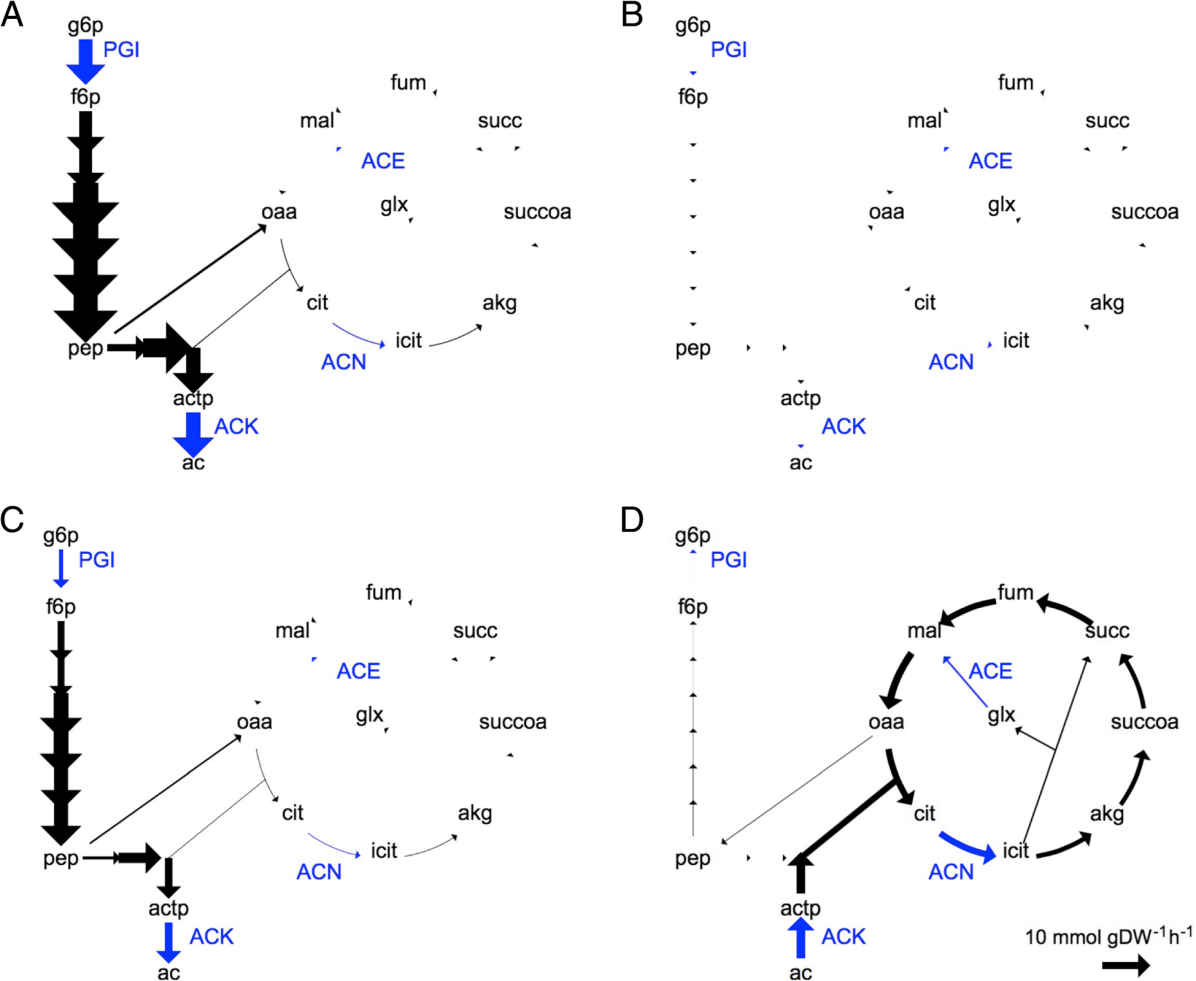
Metabolite turnover fluxes in glycolysis and TCA cycle. Fluxes in the glucose specialist (**a**, **b**) and the acetate specialist (**c**, **d**) genotypes (model IDs 12364 and 44490, respectively) during growth on glucose (**a**, **c**) and acetate (**b**, **d**). The following metabolites and reactions are shown: ac, acetate; actp, acetyl-phosphate; akg, alpha-keto-glutarate; cit, citrate; f6p, fructose-6-phosphate; fum, fumarate; glx, glyoxylate; g6p, glucose-6-phosphate; icit, isocitrate; mal, malate; oaa, oxaloacetate; pep, phospho-enol-pyruvate; succ, succinate; succoa, succinyl-coenzyme a. PGI, ACN, ACE, and ACK are the reactions catalyzed by glucose-phosphate isomerase, aconitate hydratase, malate synthase, and acetate kinase, respectively (shown in *blue*). Thickness of the *arrow* indicates the flux over the given reaction; the reference *arrow* at the *bottom right* shows a flux of 10 mmol/gDW/h

We then examined the metabolic fluxes for the two model organisms when growing on glucose and acetate (Fig. 4). On glucose, both the glucose and acetate specialists displayed similar behaviours, using the TCA cycle only partially and the glyoxylate shunt not at all (Fig. 4a and c). After switching to acetate consumption (which the glucose specialists could not do), the acetate specialists showed very different fluxes, with reverse glycolysis and full use of the TCA cycle including the glyoxylate shunt (Fig. 4b and d). We emphasize that the emergence of cross-feeding model organisms and their associated fluxes in the evoFBA simulation represents an idealized evolutionary stable state given the assumptions of the evoFBA framework.

### Adaptive diversification in one LTEE population, matching evoFBA predictions

Two distinct lineages had emerged in one of the LTEE populations, called Ara-2, by 6500 generations, and they have coexisted ever since [26, 50]. The lineages are called S (small) and L (large) after their colony sizes on agar plates. The maintenance of this polymorphism depends on a cross-feeding interaction in which the L type is a better competitor for the exogenously supplied glucose and the S type is better at using one or more secreted byproducts [26], although the precise ecological and metabolic mechanisms are still unknown. Therefore, we used predictions from the evoFBA simulations to generate hypotheses about these mechanisms.

We hypothesized that, first, L specializes on glucose and secretes acetate and, second, S specializes by improved acetate consumption. We tested this hypothesis by analyzing two evolved clones sampled at generation 6500 from the S and L lineages, named 6.5KS1 and 6.5KL4, respectively. HPLC analyses confirmed the presence of acetate in a 24-h supernatant of 6.5KL4 that was grown in the same medium as the LTEE (see Methods). Acetate was not detected after growing 6.5KS1 in that supernatant (Additional file 1: Figure S1). We then measured the acetate and glucose concentrations over time in cultures of the ancestor, 6.5KS1, and 6.5KL4 clones in DM250-glucose medium (Fig. 3b). Both the L and S clones consumed glucose faster than the ancestor, consistent with previous assays [53]. Moreover, in agreement with the evoFBA results, 6.5KL4 secreted acetate, with its concentration remaining high for many hours in the monoculture, and 6.5KS1 drew down its own acetate secretion much faster than both 6.5KL4 and the ancestor. After exhausting the glucose by 6 h, 6.5KS1 showed diauxic growth and consumed acetate until it was depleted after 9 h, whereas 6.5KL4 had barely, if at all, begun to consume acetate at that time even as it had exhausted the glucose by 5 h (Fig. 3b). These results support the hypothesis that the stable coexistence of S and L depends on acetate cross-feeding, with acetate production by both the L and S lineages and more efficient acetate scavenging by the S lineage, which exhibits a faster metabolic switch from glucose to acetate (Additional file 2: Figure S2).

### Physiology and fluxes in S and L clones agree qualitatively with evoFBA

**Fig. 5:**
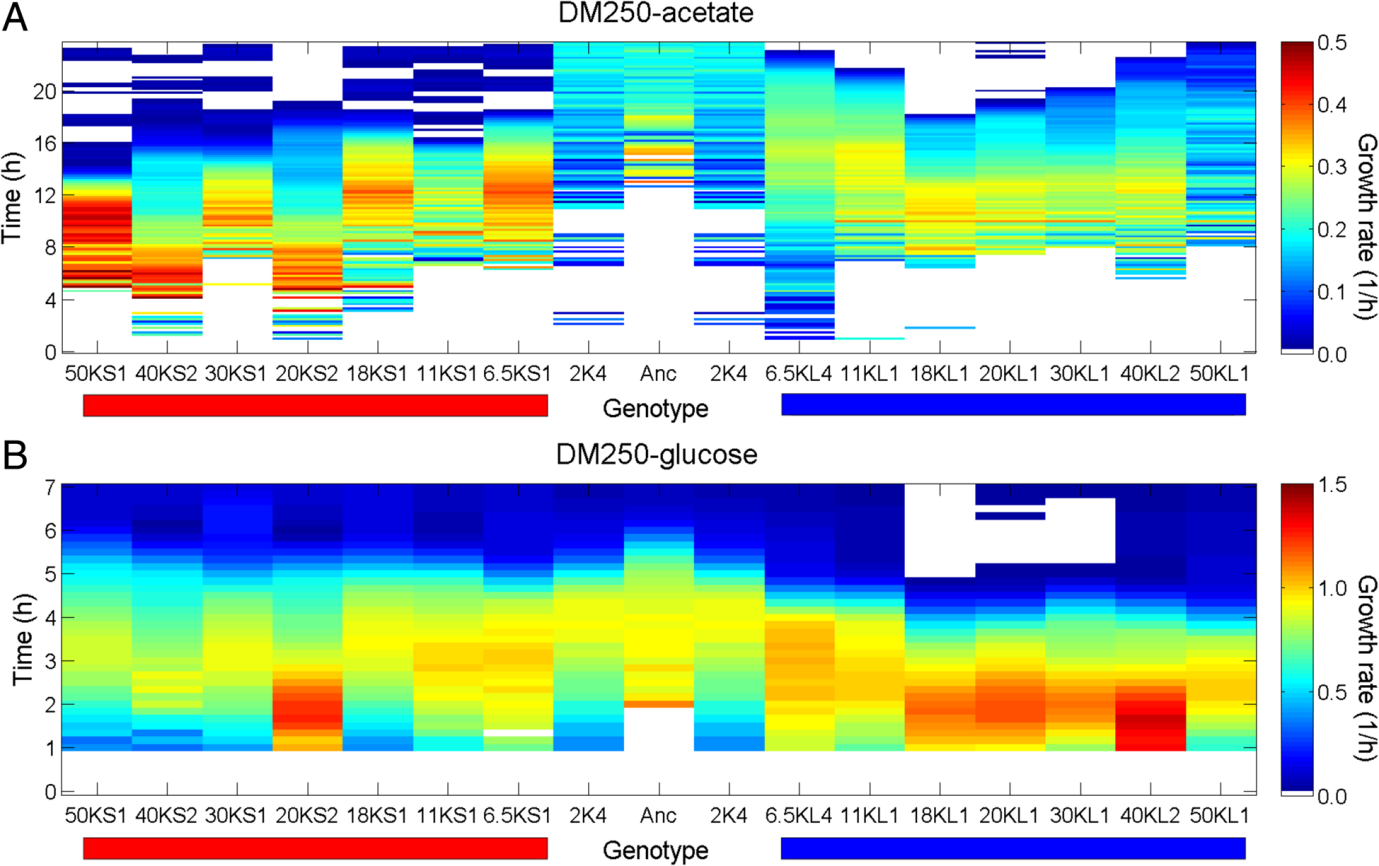
Changes in growth rates of S and L on glucose and acetate over evolutionary time. Growth of S and L clones sampled at multiple generations of the LTEE was followed in DM250-acetate (**a**) and DM250-glucose (**b**) media. Clone names are shown above the horizontal red and blue bars, which denote S and L clones, respectively. The ancestor (Anc) and a 2000-generation clone (2 K4) isolated prior to the divergence of the S and L lineages are also included. Growth rates (1/h) are shown according to the colour scale for 1-h sliding windows over 24-h and 7-h periods in the acetate and glucose media, respectively. Empty cells indicate missing values based on filtering negative rates or unreliable values (see Methods)

The evoFBA simulation reaches an evolutionary equilibrium, whereas the interaction between the S and L lineages remained highly dynamic over thousands of generations [26]. Therefore, we examined the metabolic divergence of the S and L lineages over the course of the LTEE. We first measured the ability of clones from earlier and later generations to grow in minimal media containing glucose or acetate. S clones from later generations typically grew faster and with a shorter lag phase on acetate and more slowly on glucose than S clones from earlier generations, while the opposite trends were observed in the L lineage (Additional file 3: Figure S3) (in line with previous observations [53]). Compared to the ancestor, S clones improved their growth on acetate over evolutionary time, while L clones initially improved somewhat but were variable, with the 50,000-generation L clone showing weak growth similar to the ancestor (Fig. 5). On glucose, the opposite trend was observed with L clones consistently improving compared to the ancestor, while S clones improved initially but declined in later generations (Fig. 5). These patterns of growth relative to the ancestor are consistent with previous assays using the LTEE clones [53, 54]. These evolutionary trajectories of growth on acetate and glucose indicate character displacement and suggest tradeoffs that prevent the simultaneous optimization of growth on both carbon sources. The trajectories are qualitatively consistent with the evoFBA simulations, although the evoFBA predicts complete specialization on glucose without any acetate consumption. This evoFBA prediction represents a potential evolutionarily stable end point, which might eventually occur in the S and L lineages after more generations.

**Fig. 6:**
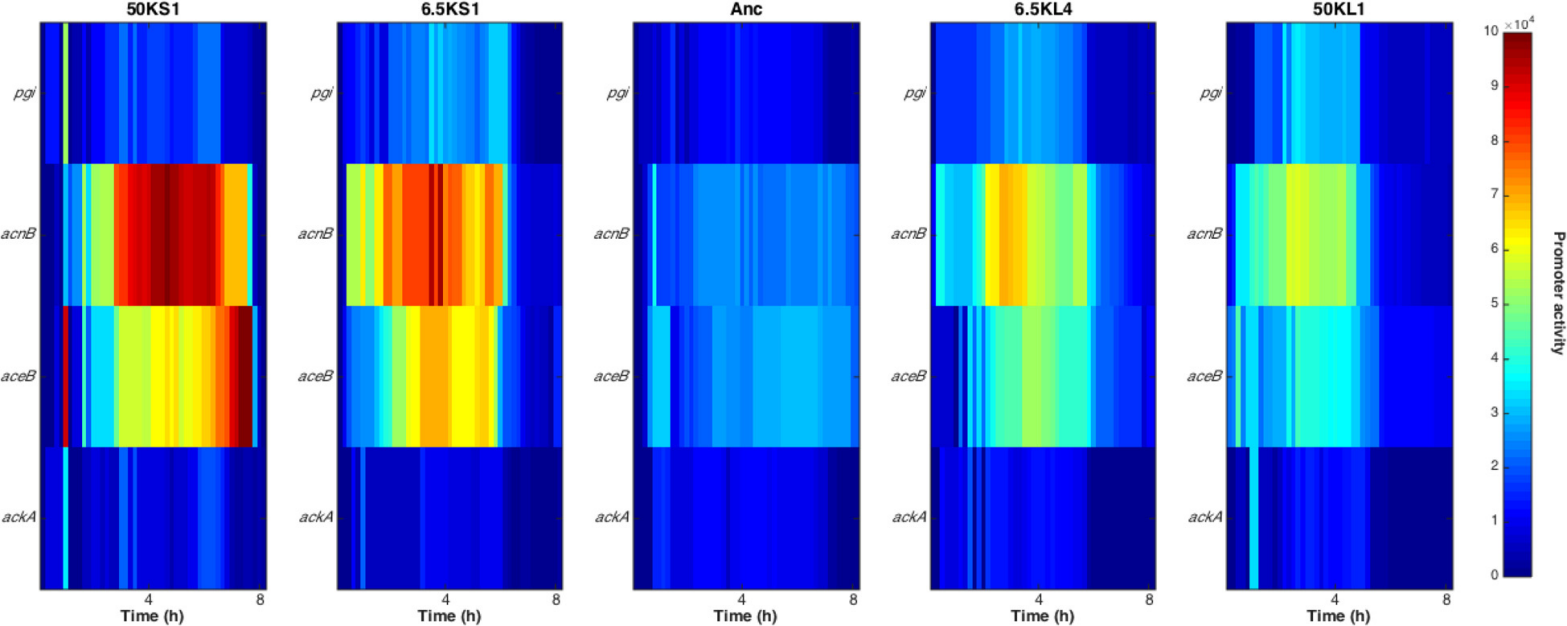
Transcription levels of four genes encoding metabolic enzymes in the ancestor and evolved clones. Promoter activities measured as (dGFP/d*t*)/OD_450nm_ for genes involved in glucose and acetate metabolism during the first 8 h of growth in DM250-glucose. The clones are, from left to right: 50KS1, 6.5KS1, ancestor (Anc), 6.5KL4 and 50KL1. The genes are, from top to bottom: *pgi* encoding glucose phosphate isomerase, *acnB* encoding aconitate hydratase, *aceB* encoding malate synthase A, and *ackA* encoding acetate kinase. Activity values are means based on three-fold replication of each assay

We then tested the flux patterns predicted by evoFBA (Fig. 4) by measuring, in several LTEE clones, the promoter activities of genes encoding four key metabolic enzymes, using transcriptional fusions with the *gfp* reporter gene (see Methods). Both S and L clones showed moderately increased promoter activity for *pgi* relative to the ancestor (Fig. 6). Both S and L clones exhibited larger increases in the promoter activities of *acnB* and *aceB* relative to the ancestor, with the S clones showing much greater increases than the L clones, consistent with the possibility of greater flux through the TCA cycle and glyoxylate shunt in the S acetate specialists. There were no obvious changes in the promoter activities of *ackA* in either the S or L lineages. Of course, there may be discrepancies between promoter activities and actual enzyme activities [55, 56]. Nonetheless, these patterns agree reasonably well with the flux predictions from the evoFBA simulations, especially as they relate to the higher activities in the S lineage of the genes that specifically promote growth on acetate. As noted above, we reiterate that the evoFBA simulations predict an eventual complete loss of the acetate-specific activities in the L lineage, whereas thus far they are merely expressed at a lower level in the L lineage than in the S lineage.

## Discussion

We developed a modeling framework, called evoFBA, which combines metabolic models that are amenable to FBA with an evolutionary algorithm to simulate the interplay of evolutionary and ecological dynamics in systems with multiple strains or species. We applied evoFBA to the LTEE with *E. coli* and predicted the emergence of two stably coexisting lineages with distinct metabolic flux distributions that promote a cross-feeding interaction. These predictions fit with the polymorphism seen in the Ara–2 population, where two lineages emerged early in the LTEE that have now coexisted for tens of thousands of generations [26, 50]. The evoFBA simulations enabled us to hypothesize specific ecological and physiological mechanisms that generate and sustain this polymorphism, and we then tested these hypotheses using the bacteria from that LTEE population. On balance, we found that the ecological, physiological, and metabolic properties of these coexisting lineages agree reasonably well with the predictions of the evoFBA.

Only one of the 12 LTEE populations evolved a persistent polymorphism that has been studied in such detail [26, 50]. However, other LTEE populations show evidence of negative frequency-dependent fitness, deep phylogenetic divergence, or both, which are consistent with adaptive diversification [57–60]. These results suggest that other populations may have evolved cross-feeding interactions similar to the one studied here, even if they were not always so persistent [59]. One possible explanation for why persistent polymorphisms did not evolve in the other populations is that the establishment of the S lineage appears to have involved epistatic interactions between multiple mutations [50], which may have limited its evolutionary accessibility [58]. This possibility reflects one of the limitations of evoFBA, which cannot capture all of the intricacies of biological evolution but instead predicts optimal end states that emerge given the simplifying assumptions of this approach. For example, mutations in evoFBA affect the rates of resource uptake, but not the rates of internal reactions in the model. This limitation reflects the computational burden of simulating a multitude of mutant genotypes, the number of which would increase greatly if all reaction rates were subject to mutation. This limitation could be relieved by the development of more efficient algorithms (allowing mutations to affect all reactions in the model), but the final evolved model organisms might not differ functionally from those based on the current approach because changes in uptake rates can already affect downstream flux distributions. Another limitation of the evoFBA approach at this time is the assumption that constraints on the uptake fluxes can be changed only by mutation, while the optimization of fluxes within those constraints is immediate through FBA [32]. In other words, metabolic fluxes change within physiological limits without delay, whereas changing the limits themselves requires mutations. These assumptions are reasonable starting points for incorporating evolutionary dynamics into an FBA framework, but physiological delays in metabolic adjustments are also sometimes important [61, 62]. Expanding the evoFBA framework to include the dynamics of physiological transitions could start by integrating previous work on incorporating gene regulation into FBA [63, 64].

Adaptive diversification is expected, and has occurred, in other evolution experiments besides the LTEE, such as when two exogenous carbon sources are provided [27, 65] and in high-glucose chemostats, where substantial acetate is produced [21, 23, 25]. However, the adaptive diversification observed in the LTEE was unexpected owing to the presence of a single carbon source, glucose, which was supplied at a low concentration [52]. Using the evoFBA framework, we predicted that acetate secretion was the primary metabolic driver leading to the emergence of the polymorphism, and that prediction was supported by our experiments. The long duration of the LTEE—including several thousand generations to establish the S and L polymorphism [26] and its persistence for tens of thousands of generations [60]—may have facilitated adaptive divergence under these more restrictive ecological conditions, in comparison with other studies of much shorter duration where glucose, acetate, or both were supplied exogenously at high concentrations [23, 25, 45]. In fact, low levels of acetate, as observed in our study, have previously been reported to favor generalists as opposed to divergence into coexisting specialists [10]. Nonetheless, the L lineage evolved higher glucose uptake rates, which led to acetate overflow and the construction of a new niche that benefited the S lineage, as occurred in the evoFBA simulations. Thus, niche construction by the bacteria led to the emergence of this polymorphism, in contrast to experiments where both carbon sources were added to the medium [45]. Despite the differences between the LTEE and previous evolution experiments [45, 65], similar metabolic processes emerged.

## Conclusion

The combination of evoFBA and experimental evolution provides a useful approach that can give insights into general mechanisms involved in the emergence of bacterial diversity and community construction. This approach may stimulate the development of even more detailed and integrated studies aimed at predicting the outcomes of evolution experiments and dynamics in multi-species systems including synthetic microbial communities [51].

## Methods

### Evolutionary flux balance analysis (evoFBA)

In each evoFBA simulation, stoichiometric metabolic models were used to simulate clonal populations with distinct genotypes. Each genotype was represented by a metabolic model, which was simulated in a dynamical FBA formalism [46] to evaluate its growth and metabolic flux rates over time. At each time step of the dynamic FBA, the metabolic model was optimized using linear optimization and a pseudo-reaction representing biomass as the objective function [66]. This optimization thus maximized growth rate given the constraints on uptake rates, i.e. it optimized biomass yield per substrate [67]. Instead of defining specific uptake rates for a particular set of media components (as in standard FBA studies), we assumed a global constraint for all carbon and oxygen uptake reactions in each model organism. By limiting total uptake in the model (including O_2_ “uptake”), we represent cellular limitations that can arise from many different factors, including redox cycling [20, 47], respiratory chain [18], enzyme expression [16, 48], and substrate uptake [17]. Similar implementations of global constraints in FBA models have been employed previously to study diauxic shift and substrate preference in *E. coli* [18, 19, 37]. The global uptake constraint implemented here favored a minimization of fluxes given the maximization of the FBA objective in order to achieve the most efficient use of cellular resources for growth [34].

New model organisms were generated by random mutation from existing ones. Mutations altered specific bounds on individual uptake rates, while maintaining an overall total flux constraint of carbon and oxygen into the model organism. Thus, mutations change how the overall uptake flux is distributed across different substrates, and they allow a second level of optimization to occur over evolutionary time in addition to the optimization that occurs by FBA over the physiological time scale. Focusing evolution on a subset of reactions made computation of the ecological and evolutionary dynamics feasible; even so, the simulations presented here took over 20 days on a dedicated high-performance computer to simulate over 90,000 different model organism genotypes and their associated population and metabolite dynamics. A more complete simulation might encompass genome-scale models with evolution of all reactions in the model and with global constraints on total protein biomass [19] and membrane space [18]. The current implementation of evoFBA was unable to perform such simulations in a reasonable timeframe and with appropriate numbers of replicate simulations; efforts to run evoFBA with mutations allowed for all reactions caused a slowdown of over 10-fold relative to the current implementation.

For the evoFBA simulations, we implemented the *in silico* equivalent of the LTEE with *E. coli*. The simulations started with a population comprised of a single model genotype that represented the central metabolism of *E. coli* [68]. This model included 95 reactions, 75 metabolites, and 20 exchange reactions. The uptake of nutrients from the medium (i.e., the flux over the exchange reactions) was simulated by a Michaelis-Menten function for each substrate, *v*_*j*_, as follows:

1 $$ {v}_j=\frac{v_{max,j}\cdot \left[{S}_j\right]}{K_m+\left[{S}_j\right]} $$

where *v*_*max,j*_ is the maximum uptake rate of the *j*th substrate in millimoles per g dry weight per h (mmol/gDW/h), [*S*_*j*_] is the concentration of the *j*th substrate in mmol/l, and *K*_*m*_ is the half-saturation constant of the transporter in mmol/l. For simplicity, we arbitrarily set the initial *K*_*m*_ values for all uptake reactions to 0.01 mmol/l. The *v*_*max,j*_ values were allowed to evolve by mutation (see next section below). The value of *v*_*j*_ was then used as the uptake bound for the exchange reaction of each substrate when running FBA. For each simulated day, we evaluated each model using dynamic FBA [46] over the course of 24 h with 1-min steps; the simulation used a 10-ml batch reactor, as in the LTEE. At each time step, we set the *v*_*j*_ values for each model using Eq. 1, used FBA to determine growth rate, and updated the biomass as follows:

2 $$ B{M}_{t+1,i}=B{M}_{t,i}\ \left(1 + \frac{\mu_i}{ln(2)\cdot 60}\right) $$

where *BM*_*t,i*_ is the biomass in gDW of the clone represented by the *i*th model genotype at time *t*, and *μ*_*i*_ is the growth rate of that model computed by FBA [29]. After updating the biomass of all model genotypes, the resulting concentration of each substrate was reset as follows:

3 $$ {\left[S\right]}_{j,t+1}={\left[S\right]}_{j,t}-\kern0.5em {\displaystyle \sum_i}\frac{B{M}_{i,t}\cdot {v}_{j,i}}{60\cdot V} $$

where [*S*]_*j,t*_ is the substrate concentration of the *j*th substrate at time *t* (mmol/l), *BM*_*i*_ is the biomass of the *i*th model clone at time *t* (gDW/l), *v*_*j,i*_ is the uptake rate of the *j*th substrate by the *i*th model genotype as computed by FBA (mmol/gDW/h), and *V* is the culture volume (l). To mimic the LTEE*,* we started each day’s culture with glucose at 0.1389 mmol/l. The culture was started with one model genotype (i.e., the core *E. coli* model [68]) having a *v*_*max*_ for glucose and acetate of 10 mmol/gDW/h each, and for oxygen of 20 mmol/gDW/h, i.e. the total uptake constraint was set at 40 mmol/gDW/h based on previous values for the combined uptake of carbon and oxygen [67]. The individual uptake rates for 14 carbon sources represented in the *E. coli* core model (acetate, acetaldehyde, α-ketoglutarate, ethanol, formate, fructose, fumarate, glucose, glutamine, glutamate, lactate, malate, pyruvate, succinate) and oxygen were subject to mutation in evoFBA. The exchange rates for phosphate, ammonia, water, protons, and carbon dioxide had no limits, reflecting the fact that carbon is the growth-limiting factor in the LTEE.

### Representing mutations in evoFBA

The point mutation rate of *E. coli* in the LTEE (excluding populations that evolved mutator phenotypes [60]) has been estimated to ~10^−10^ per base pair per generation, which equals ~4 × 10^−4^ per genome per generation [69]. Directly mapping mutations from bacteria to evoFBA model organisms is not possible. The mutable “genome” in the model organism has only 15 targets, as opposed to thousands of genes and millions of base pairs in an *E. coli* genome. Owing to these differences and computational limitations, we introduced mutations at the rate of 10^−6^ per model cell per generation in evoFBA. During the simulations, mutations were introduced into the population of each model genotype at each time step (i.e., simulated minute) according to the number of cells produced in that step and those expected to contain a mutation (*N*_*m(i)*_) as follows:

4 $$ {N}_{m(i)} = \frac{N_i\ {\mu}_i}{ln(2)\cdot 60 \cdot {10}^6} $$

where *N*_*i*_ is the population size of model organism *i. N*_*i*_ was calculated from the biomass of model clone *i* divided by the mass of one cell in gDW; we used a mass of 600 fg/cell, which was reported previously for exponentially growing *E. coli* cells [70]. When *N*_*m(i)*_ was between 0 and 1, *N*_*m(i)*_ was used as a probability to determine whether or not a mutant was introduced; when *N*_*m(i)*_ was ≥1, a single mutant was always introduced.

As noted, each genotype in evoFBA corresponds to a different stoichiometric model with associated *v*_*max*_ values. When a mutation occurred, one of the uptake reactions was chosen at random and the maximum rate for that reaction was changed as follows:

5 $$ {v}_{max,m, new} = {v}_{max,m, old} + a $$

where *v*_*max,m,new*_ and *v*_*max,m,old*_ are the new and old rates for the mutated reaction *m*, and *a* is a random number from the uniform distribution over the interval ( −10,10). Each individual uptake rate was further constrained to lie between 0 and 40, such that the total uptake rate of 40 mmol/gDW/h was not violated. After any mutation, all other uptake reactions were updated as follows:

6 $$ {v}_{max,j,\  new}=\frac{v_{max,\ j, old}}{{\displaystyle {\sum}_k}{v}_{max,k, old}}\left(40,, -,, {v}_{max,m, new}\right) $$

where *k* includes all uptake reactions except the mutated one. This adjustment ensures a constant total uptake flux across the membrane of 40 mmol/gDW/h. This mutation scheme generates strong tradeoffs between uptake reactions. The large effects of the mutations on reaction rates were chosen for computational speed of the evolutionary simulations; additional simulations with smaller maximum mutation steps produced qualitatively similar results (Fig. 2).

### Simulating serial transfer and selection

To simulate the LTEE’s daily transfer cycles [2], we used dynamical FBA to compute growth over 24 h; selection is a direct consequence of the differential growth of the model genotypes. After 24 h, a dilution was performed by randomly drawing 1 % of the model organisms, which constituted the initial population for the next simulated day. The next day’s medium included 99 % of the initial medium and 1 % of the spent medium from the end of the previous day. The simulated growth and dilution ran for a total of 550 cycles. Results from replicate simulations (Fig. 2) are qualitatively similar to those in Fig. 1. The population dynamics arising from these simulations are expected to give rise eventually to one dominant clone in each stably coexisting lineage. However, similar model organisms may occur within a simulation as a result of independent mutations before any one of them has reached its population maximum (e.g., model genotypes 13437, 12364 and 12719 in Fig. 1). However, if the model organisms differ even slightly in their uptake rates, then one genotype should eventually prevail through competitive exclusion, unless the model organisms occupy distinct ecological niches (Fig. 1).

### Computation

Simulations were performed using MATLAB (Math Works, Natick, Massachusetts) and dynamic FBA calculations using the COBRA toolbox [71]. The MATLAB scripts used to run evoFBA and analyze the data are freely available at [72].

### LTEE and bacterial strains

The LTEE consists of 12 populations founded from the same ancestral strain of *E. coli*, REL606 [73], that have been propagated since 1988 by daily 1:100 dilutions in Davis minimal medium [52] supplemented with glucose at 25 mg/l (DM25). Here, we focused on one population, called Ara–2, in which two lineages, S and L, diverged before 6500 generations and have co-existed ever since [26, 50, 60]. We studied the ancestor and one clone sampled from each lineage at 6500, 11,000, 18,000, 20,000, 30,000, 40,000 and 50,000 generations. Each evolved clone is named by its generation followed by S or L according to its lineage and an arbitrary numeral for a given clone. For example, 6.5KS1 is a clone from the S lineage that was sampled at 6500 generations.

### Media and culture conditions

Bacteria were grown in the same medium as used in the LTEE [52], except that the carbon source was glucose at 250 mg/l (DM250-glucose), glucose at 1000 mg/l (DM1000-glucose), or acetate at 250 mg/l (DM250-acetate). These higher concentrations were used to increase cell density and thereby improve the accuracy of measurements of cell growth (e.g., Fig. 5) and concentrations of residual resources and secreted metabolites (e.g., Additional file 1: Figure S1). After overnight growth in DM1000-glucose, strains were inoculated by a 10,000-fold dilution into DM250-glucose, where they grew for 24 h at 37 °C with shaking at 120 rpm as an acclimation step. For each strain, three replicate acclimation cultures were then inoculated as duplicates, each at a 1:100 dilution, into DM250-glucose or DM250-acetate and incubated in 96-well microtiter plates at 37 °C for 24 h. Growth was monitored using an Infinite M200 microplate reader (Tecan, Lyon, France) by measuring the OD_450nm_ every 10 min. Growth rates were computed from filtered OD data as dln(OD_450_)/d*t* over a sliding window of 1 h, using MATLAB. We report the mean of the three replicates. Filtering was performed by removing negative growth rates and mean growth rates that were more than 0.2 units above or below the immediately adjacent data points (outliers).

### Measuring glucose and acetate concentrations

The ancestor, 6.5KS1, and 6.5KL4 clones were grown in DM250-glucose as described. Samples were taken at time 0 and every h for 9 h. After centrifugation to remove cells, we measured glucose and acetate concentrations in the supernatant using the Glucose Assay Kit (Merck Millipore, Lyon, France) and Acetic Acid Assay Kit (Megazyme, Pontcharra-sur-Turdine, France), respectively, following the manufacturers’ recommendations.

### Analysis of flux patterns in individual model organisms

We simulated the growth of the evoFBA model organisms with IDs 44490 and 12364 (Fig. 1) to obtain the flux values for their biochemical reactions. Each model organism was simulated using dynamical FBA in medium containing 0.1389 mM glucose, the same concentration as in the LTEE. Each simulation ran for ten 24-h periods with daily 1:100 dilutions; the last day was used to record the flux values, in order to remove any effect of the initial conditions. The flux patterns for growth on glucose were taken 10 min after the onset of growth (Fig. 4a and c), and for growth on acetate at 388 min because glucose was exhausted while acetate was still present at a substantial level (Fig. 4b and d). From the flux patterns, we identified several reactions of interest that showed differences between the two evolved model organisms (highlighted in blue in Fig. 4).

### Identification of metabolites in filtrates of spent cultures of 6.5KL4

We analyzed by HPLC and GC-MS the metabolic by-products secreted by clone 6.5KL4 using filtrates from 24-h spent cultures of that clone in DM25- and DM250-glucose, both before and after growth of clone 6.5KS1. For HPLC, 1 ml of filtrate was acidified with 5 μl 1 M H_2_SO_4_, incubated at room temperature for 5 min, and passed through a 0.45-μm regenerated cellulose syringe filter (PHENEX RC Membrane, Phenomenex, Le Pecq, France). Samples were then analyzed on an Agilent 1260 Infinity HPLC system equipped with a Rezex ROA-Organic Acid (8 %) 300 × 7.8-mm column (Phenomenex) and a diode array detector. The analytical conditions were as follows: mobile phase, 5 mM H_2_SO_4_; flow rate, 0.6 ml/min; column temperature, 35 °C; injection volume, 50 μl; wavelength scan range, 190–400 nm; detection wavelength, 210 nm; and run time, 35 min. Concentrations of acetate and fumarate in the L-clone filtrates were determined from linear standard curves over the ranges of 0–10 mM and 0–100 μM, respectively, and with lower detection limits of 0.1 mM and 0.3 μM, respectively. Succinate, lactate, formate, propionate, and butyrate can also be separated under these analytical conditions with detection limits similar to acetate, but they were not detected in any samples.

GC-MS analysis of volatile compounds was performed using an Agilent GC HP6890 gas chromatograph equipped with a Varian CP-WAX 58 column (length, 25 m; internal diameter, 0.25 mm; film thickness, 0.20 μm), and coupled to an MSD5973 mass sensitive detector. The sample (600 μl) was cooled on ice, acidified with 50 μl 4 M HCl, and extracted with 0.375 g NaCl and 650 μl ice-cold ether. After vortexing three times for 10 s each, with 30 s cooling intervals, the sample was centrifuged for 5 min at 10,000 rpm and placed on ice for 5 min. The upper organic layer (2.5 μl) was then injected manually into the GC using an ice-cold syringe (injection in split mode, split ratio = 10). The column was held at 40 °C for 1 min, ramped to 200 °C at a rate of 5 °C/min, and held for a further 3 min, giving a total run time of 36 min. The solvent delay for the MSD was 1.4 min and the mass scan range was set to 35–300 atomic mass units. The presence of acetate (retention time 12.2 min) in the L filtrate after growth in both DM25- and DM250-glucose was confirmed by this method. The estimated concentration from the DM250-glucose filtrate was 510 μM, which is close to the 480 μM detected by HPLC (Additional file 1: Figure S1). The concentration of acetate in the L filtrate from DM25-glucose was about one-tenth that detected in DM250-glucose. Ethanol (retention time 2.1 min) was also detected in the filtrates of all three strains tested (ancestor, 6.5KL4, and 6.5KS1). Other metabolites including isopropanol, butanol, acetoin, acetone, formic acid, propionic acid, butyric acid, isobutyric acid, and valeric acid were not detected (with lower detection limits around 50–100 μM in scan mode).

### Analysis of promoter activities in LTEE clones

We measured the activities of the promoters of four genes—*pgi*, *acnB*, *aceB*, and *ackA—*that encode enzymes associated with reactions of interest (Table 1) given the results of evoFBA (Fig. 4). We used the corresponding reporter plasmids from the *E. coli* library of *gfp* transcriptional fusions [74]. Each of the four plasmids, as well as the empty pUA66 reference plasmid, was introduced into the ancestor, 6.5KS1, 6.5KL4, 50KS1, and 50KL1 clones. Each plasmid-bearing clone was grown in DM250-glucose supplemented with 25 μg/ml kanamycin. Both OD_450nm_ and GFP fluorescence were measured every 10 min for 24 h in the microplate reader. Promoter activities were estimated as the rate of GFP production from the promoter region [74]. They were computed using MATLAB as (dGFP/d*t*)/OD_450nm_, where GFP is the fluorescence signal after subtracting the value for the empty plasmid and division by OD_450nm_ standardizes the data with respect to cell biomass density. We show the mean values from three replicate experiments (Fig. 6).

**Table 1:** Genes used in the analysis of promoter activities

Name	Gene ID	Gene	Protein	FBA model term
Glucose-phosphate isomerase	948535	*pgi*	PGI	PGI
Aconitate hydratase	944864	*acnB*	ACN	ACONTb
Malate synthase A	948512	*aceB*	ACE	MALS
Acetate kinase	946775	*ackA*	ACK	ACKr

## Additional files

Additional file 1: Figure S1. HPLC profiles of filtrate from spent cultures of 6.5KL4 before and after growth of 6.5KS1. Partial HPLC chromatograms, scaled in milli Absorbance Units (mAU) at 210 nm, showing elution time (min) of key metabolites for the filtrate of a 24-h spent culture of clone 6.5KL4 in DM250-glucose (A), and for the same filtrate after 24 h of growth of clone 6.5KS1 at 37 °C (B). The L filtrate contained 2-hydroxyglutarate, acetate, and fumarate. The S clone consumed the acetate and fumarate, but not the 2-hydroxyglutarate. The acetate peak indicates a concentration of 480 μM, whereas the fumarate peak indicates a concentration of only 0.67 μM; the molar absorption coefficient of fumarate at 210 nm is more than 300 times greater than that of acetate. (PDF 64 kb) Additional file 2: Figure S2. Diauxic growth in DM250 medium containing glucose and acetate at 10:90 ratio. Clone 6.5KS1 (red) exhibits a diauxic shift from glucose to acetate consumption much earlier than either the ancestor (green) or clone 6.5KL4 (blue). Curves show the average of three biological replicates. (PDF 113 kb) Additional file 3: Figure S3. Growth curves of the ancestor and evolved clones in DM250-glucose and DM250-acetate media. **A** Growth curves of the Anc and pre-divergence clone 2 K4 (both shown in green) on glucose (left) and acetate (right). **B** Growth curves of S (red) and L (blue) clones sampled at seven generations (6.5, 11, 18, 20, 30, 40, and 50 K arranged chronologically from top to bottom) on glucose (left) and acetate (right). In each panel, curves show the average (heavy line) of 3–6 replicate assays (lighter lines) for each clone; curves for individual replicates are not always visible when they are close to the mean or other replicates. (PDF 186 kb)
