# Supplementary figures and images for: Metabolic modelling in a dynamic evolutionary framework predicts adaptive diversification of bacteria in a long-term evolution experiment
Source: BMC Evol Biol. 2016 Aug 20;16:163. doi: 10.1186/s12862-016-0733-x (PMC4992563; doi:10.1186/s12862-016-0733-x)

A

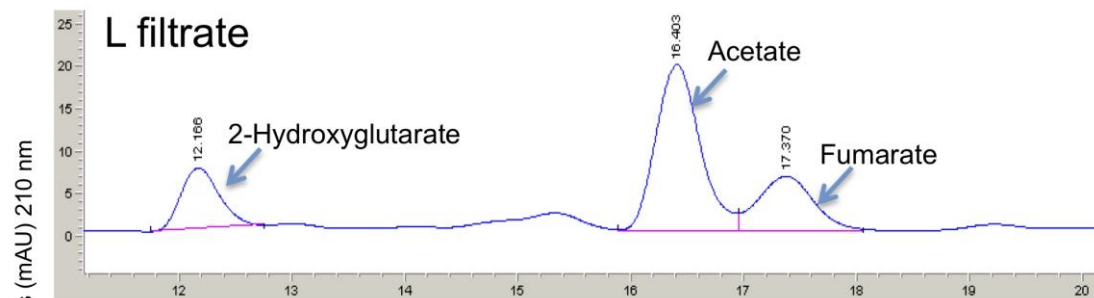

B

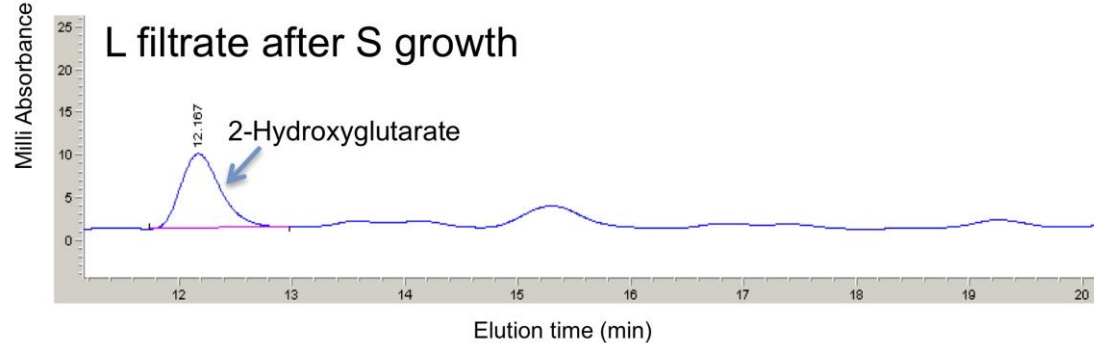

Supplement: Additional file 1: Figure S1. — HPLC profiles of filtrate from spent cultures of 6.5KL4 before and after growth of 6.5KS1. Partial HPLC chromatograms, scaled in milli Absorbance Units (mAU) at 210 nm, showing elution time (min) of key metabolites for the filtrate of a 24-h spent culture of clone 6.5KL4 in DM250-glucose (A), and for the same filtrate after 24 h of growth of clone 6.5KS1 at 37 °C (B). The L filtrate contained 2-hydroxyglutarate, acetate, and fumarate. The S clone consumed the acetate and fumarate, but not the 2-hydroxyglutarate. The acetate peak indicates a concentration of 480 μM, whereas the fumarate peak indicates a concentration of only 0.67 μM; the molar absorption coefficient of fumarate at 210 nm is more than 300 times greater than that of acetate. (PDF 64 kb) [file 12862_2016_733_MOESM1_ESM.pdf]

**glucose:acetate 10:90**

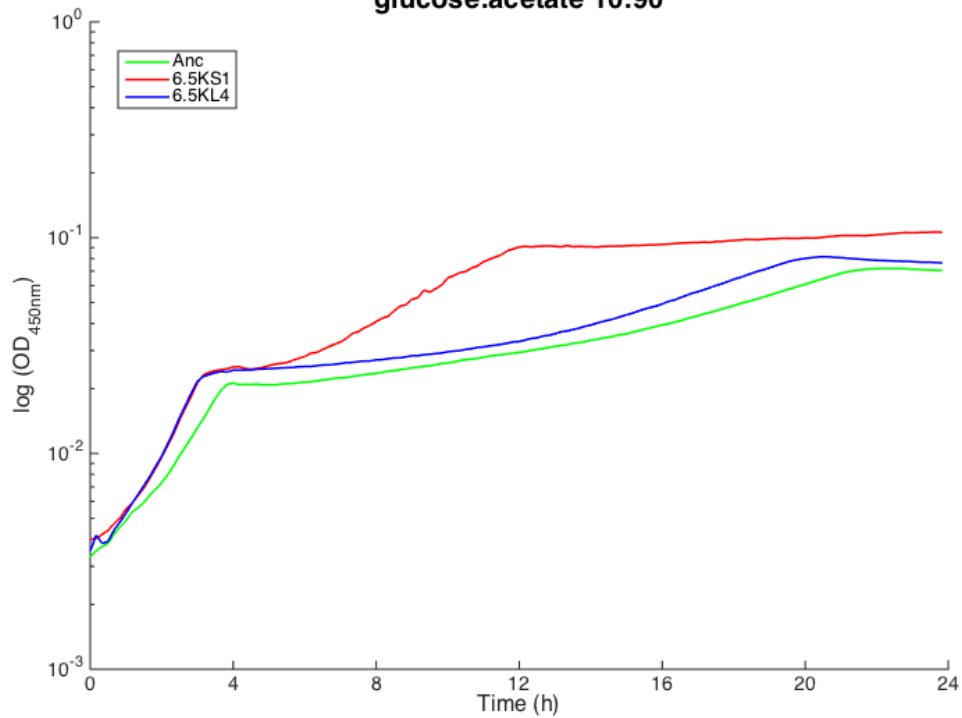

Supplement: Additional file 2: Figure S2. — Diauxic growth in DM250 medium containing glucose and acetate at 10:90 ratio. Clone 6.5KS1 (red) exhibits a diauxic shift from glucose to acetate consumption much earlier than either the ancestor (green) or clone 6.5KL4 (blue). Curves show the average of three biological replicates. (PDF 113 kb) [file 12862_2016_733_MOESM2_ESM.pdf]

# DM250-glucose

# DM250-acetate

A

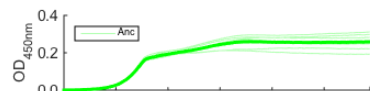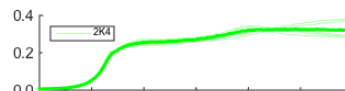

B

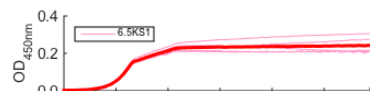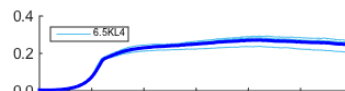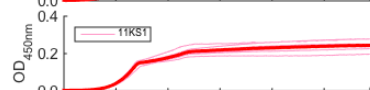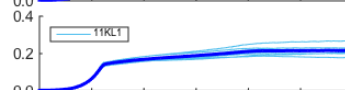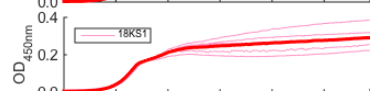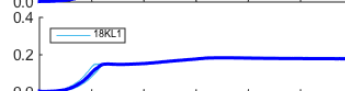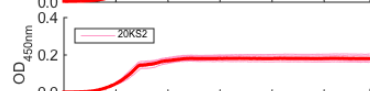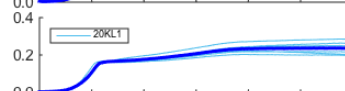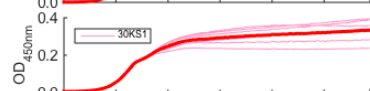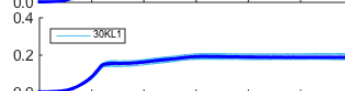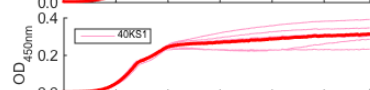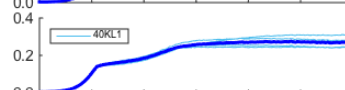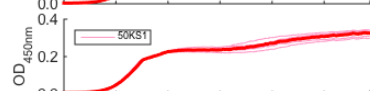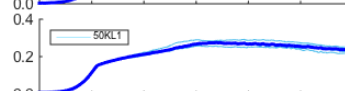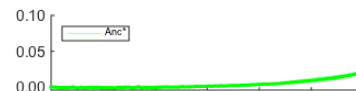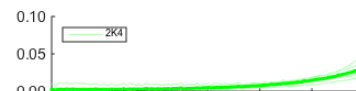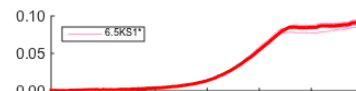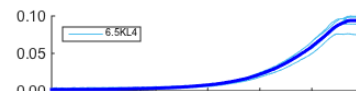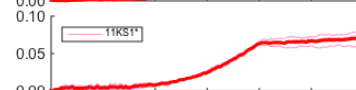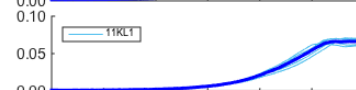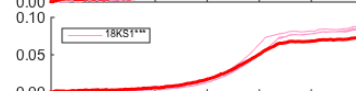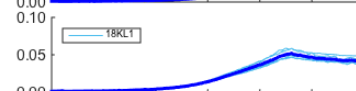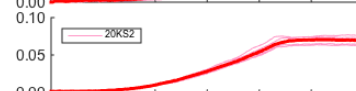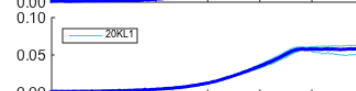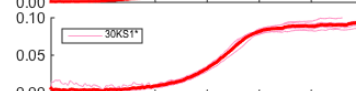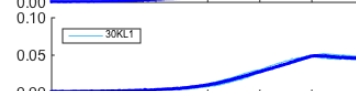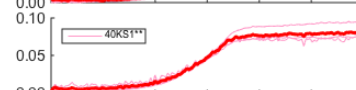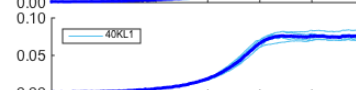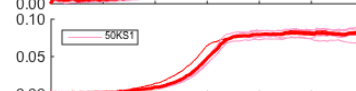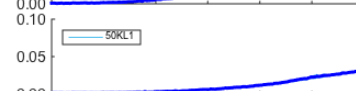

Supplement: Additional file 3: Figure S3. — Growth curves of the ancestor and evolved clones in DM250-glucose and DM250-acetate media. A Growth curves of the Anc and pre-divergence clone 2 K4 (both shown in green) on glucose (left) and acetate (right). B Growth curves of S (red) and L (blue) clones sampled at seven generations (6.5, 11, 18, 20, 30, 40, and 50 K arranged chronologically from top to bottom) on glucose (left) and acetate (right). In each panel, curves show the average (heavy line) of 3–6 replicate assays (lighter lines) for each clone; curves for individual replicates are not always visible when they are close to the mean or other replicates. (PDF 186 kb) [file 12862_2016_733_MOESM3_ESM.pdf]
